# Disorder in H^+^-irradiated HOPG: effect of impinging energy and dose on Raman D-band splitting and surface topography

**DOI:** 10.3762/bjnano.9.253

**Published:** 2018-10-19

**Authors:** Lisandro Venosta, Noelia Bajales, Sergio Suárez, Paula G Bercoff

**Affiliations:** 1 Universidad Nacional de Córdoba, FAMAF, Medina Allende s/n, Ciudad Universitaria. 5000 Córdoba, Argentina; 2 CONICET, IFEG, Medina Allende s/n, Ciudad Universitaria. 5000 Córdoba, Argentina; 3 Centro Atómico Bariloche. Av. Bustillo 9500. 8400 San Carlos de Bariloche, Argentina

**Keywords:** disorder, highly oriented pyrolytic graphite (HOPG), ion–solid interactions, Raman spectroscopy, topography

## Abstract

Disorder was induced in pristine highly oriented pyrolytic graphite (HOPG) by irradiation with H^+^ ions with energies of 0.4 MeV and 1 MeV, and doses of 10^14^ ions/cm^2^ and 10^16^ ions/cm^2^. Raman spectroscopy was used as the main technique to characterize different samples and gain new insights on the splitting of the D band into two components (D_1_ and D_2_), trying to correlate this feature of the vibrational spectrum with the impinging energy and dose. An increased *I*_D2_/*I*_G_ ratio in comparison with *I*_D1_/*I*_G_ was observed in the irradiated samples. This behavior indicates that the impinging energy mainly affects the D_1_ component, while the D_2_ component is strongly dominated by the dose. We expect a larger contribution of defects (originating from the rupture of C–C sp^2^ symmetry through the formation of C–H sp^3^ bonds) to the D_2_ component than to the D_1_ component. SQUID measurements of the irradiated samples showed an enhancement in the normalized remanence, as well as an increment in coercivity compared to pristine HOPG, consistent with H^+^-induced point-like defects as well as C–H bonds. AFM scanning after Raman and SQUID characterization showed a distribution of surface defects, which were ascribed to the burst of hydrogen blisters formed as a consequence of the irradiation process. The results presented in this work contribute to the current trend in nanotechnology in areas devoted to the control of properties by defect engineering in carbon-based materials.

## Introduction

The development of novel methods to control the properties of carbon-based materials by introducing disorder is currently a subject of interest for many nanotechnological applications [1–3]. The identification of particle-induced disorder in the sp^2^ carbon network [3–7], such as the creation and aggregation of defects and/or impurities, has been mainly conducted by using Raman spectroscopy as a fast and non-destructive tool [5,8–9]. In fact, this technique enables the characterization of a disorder signature by the observation of the Raman D band, located at ca. 1345 cm^−1^ [5,10], as well as the D′ band, located at ca. 1620 cm^−1^ [11], in addition to the characteristic Raman G and 2D bands of pristine graphite (at ca. 1580 cm^−1^ and ca. 2725 cm^−1^, respectively). However, despite the fact that extensive theoretical and experimental studies of graphene/graphite Raman spectra have contributed to elucidating the correlations between disorder and the shape of the D band [3–15], a clear explanation of the dependence of the D-band components on the irradiation parameters together with the type of defect is still a challenge.

Hydrogen-irradiated carbon allotropes have received special attention as promising materials to develop hydrogen storage devices [16–21], as well as graphane, a new sp^3^-hybridized material, based on graphene chemically modified by a hydrogenation process that leads to C–H bond terminations [17–18]. Visible Raman characterization of hydrogenated graphene reveals the rising of a D band that is remarkably sharper [17–18] than that expected for nanostructured carbon materials with structural disorder [8–9]. The prominent D band of hydrogenated graphene originates from the symmetry rupture of C–C sp^2^ bonds after the formation of C–H sp^3^ bonds [17–18].

Moreover, hydrogenated graphene showed a slight broadening and lower intensity of the 2D-band relative to G-band intensity, in addition to a band located around 2950 cm^−1^, assigned to a combination mode (D + D′) [18]. It was also found that multilayer graphene with the same top-layer defect density as single-layer graphene exhibits a lower *I*_D_/*I*_G_ ratio because intact sub-layers with π-stacking also contribute to the Raman spectrum [17–18]. On the other hand, it is worth noting that even when most of the carbon bonds in hydrogenated graphene are sp^3^-hybridized, their contribution to the Raman spectrum is not expected due to the negligible cross section of C–C sp^3^ bonds at visible-wavelength excitation [9,15,18]. Since Raman spectroscopy with visible wavelengths does not allow for a distinction of the contribution of structural and topological defects from the contribution of C–H bonds to the D band [21], especially when the H content is lower than 20% [15], the use of complementary techniques is advantageous in order to gain a better insight into the origin of defects. Atomic force microscopy (AFM) can help to reveal an increase in the graphene/graphite surface roughness, which has been correlated with the disorder generated by increasing hydrogen irradiation doses [21–23]. Furthermore, magnetic measurements are also promising as a complementary characterization technique for irradiated HOPG since it is currently accepted that a certain kind of ion-induced disorder in HOPG can give rise to uncompensated magnetic moments, in a magnitude detectable with a SQUID magnetometer (of the order of 10^−6^ emu or less [24–26]).

In previous papers, we reported a multi-characterization of HOPG with electron-induced defects [27–28]. Our Raman results showed that electron irradiation induced the appearance of the D band, an effect that was assigned to distortions in the electronic density of HOPG. Now, we intend to contribute to the understanding of structural changes in graphitic materials generated by intentional ion irradiation of HOPG surfaces. In this work we analyze the effect of dose and impinging energy of H^+^ ions on the D band of irradiated HOPG, which exhibits a double splitting. Magnetic measurements are also included, in order to correlate disorder coming from irradiation with changes in remanence and coercivity. Additionally, we show results of topographical characterization performed by AFM after vibrational and magnetic measurements. A high density of surface defects is observed, probably due to the burst of the bubbles of stored H_2_ molecules inside the HOPG matrix. This phenomenon has been reported in hydrogenated HOPG produced by other methods [21–22]. The rough morphology obtained in our irradiated HOPG samples might be used as a platform for hydrogen on-board storage, molecule pinning and other carbon-based clean-energy applications. This is a topic of current interest in nanotechnology, in areas devoted to the control of properties by defect engineering in carbon-based materials.

## Experimental

The graphite used for this work was HOPG of ZYB grade (SPI Supplies, quality SPI-2). Several pieces were cut from the as-received sample, into identical rectangles of 4 × 6 mm^2^ in order to repeat the experiments at least three times and check for reproducible results. Thus, three sets of five samples were prepared, using four of them for irradiation and one of them as a reference (pristine). Prior to any characterization, these pieces were carefully washed with acetone, to remove any possible contamination introduced during the cutting and handling [24]. According to the manufacturer, SPI-2 is very similar to ZYB. This grade exhibits a mosaic angle as small as 0.8° ± 0.2° and is slightly less ordered than ZYA. The lateral grain size can range from 0.5 mm to 1 mm, and the density of the material is 2.27 g/cm^3^.

PIXE (particle-induced X-Ray emission) measurements were performed with each piece prior to irradiation. PIXE is a useful technique for detecting contaminants, allowing for the detection of elements even when the concentrations are only a few parts per million. The measurements were performed with a low current of protons at 2 MeV in order to leave the graphite lattice fairly undisturbed prior to the ion irradiation. Our PIXE spectra (not shown) had low counting rates in the region of energies close to those of the Fe Kα and Kβ lines, indicating that there are no noticeable peaks between 6 and 7.5 keV assigned to the presence of Fe in the sample. It is worth noting that because we did not measure with standards to compare with, our measurements were only oriented to identify a possibly significant Fe contamination. Instead, we observed the presence of some other contaminants attributed to Al, coming from the collision chamber; Si, probably from the substrate where the HOPG sample was mounted and some traces of Ca, of unknown origin.

Irradiation experiments were performed using a Tandem NEC Pelletron 5SDH of 1.7 MV, at two impinging energies: 0.4 MeV (low energy, LE) and 1 MeV (high energy, HE), using two different doses: 10^14^ ions/cm^2^ (low dose, LD) and 10^16^ ions/cm^2^ (high dose, HD). These irradiation conditions were chosen in order to ensure a maximum energy transfer to recoil C atoms and optimize the defects density within this energy range. Larger energies would produce a lower amount of defects and demand a much higher dose to observe damage. Our choice of the minimum dose (LD = 1 × 10^14^ ions/cm^2^) was made to ensure a controlled quantity of impinging ions on the samples and reduce uncertainties due to beam variations. The dose imparted to the samples is proportional to the amount of deposited ions. The irradiation time was set to the current variation (*I* = *Q*/*t*) in order to keep constant the total charge accumulated in each sample. A charge of 50 μC, assures a dose of 10^16^ H^+^ ions/cm^2^, while a dose of 10^14^ ions/cm^2^ is achieved with a charge of 1 μC. The irradiation energy was chosen according to the desired penetration depth of the ions in the sample, previously calculated via numeric simulations using the software SRIM [29]. Approximate penetration depths of 3 and 12 μm were obtained for 0.4 MeV and 1 MeV ions, respectively. The four samples used for this study were labeled according to these parameters and are listed below in Table 1. The irradiation spot was approximately circular, with a diameter of ca. 1.5 mm, and was located at the geometric centre of each sample.

Several Raman spectra of each sample were measured with a laser Raman spectrophotometer (Confocal Horiba Jobin-Yvon LabRam HR). The excitation wavelength and laser power were 514 nm and (2.8 ± 0.2) mW, respectively. The laser spot diameter was 1 µm and according to this, the separation between each Raman measurement on the irradiated HOPG region (the geometrical centre of the sample) was also chosen as 1 µm, in order to have enough statistics on the defective area.

After Raman measurements, the magnetic moment as a function of the applied field was measured at 4 K with a Quantum Design SQUID with RSO, in order to accurately measure any magnetic changes in the graphite samples, which are of the order of (or less than) 10^−6^ emu. The magnetic field was applied parallel to the graphene planes to diminish the contribution of the diamagnetic background. The samples were transferred from the irradiation chamber to the SQUID holder by using a portable vacuum chamber in order to avoid contamination during manipulation.

After Raman and SQUID characterizations, atomic force microscopy (AFM) measurements were performed at room temperature using a Di-Innova Microscope (Bruker, USA) in tapping mode. Standard Si cantilevers with sharp tips were used for high-resolution topography imaging and the software Gwyddion 2.36 was used for image analyses.

## Results and Discussion

### Raman characterization

**Figure 1:**
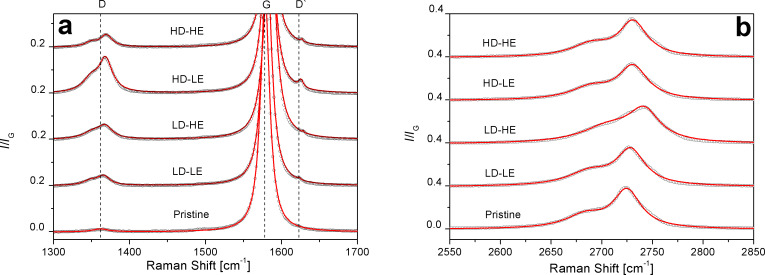
Raman spectra of irradiated HOPG (LD-LE, LD-HE, HD-LE and HD-HE). Pristine HOPG is shown for comparison. a) D band and D′ band and b) 2D band. Experimental points are shown as circles while the solid lines correspond to the fits.

Figure 1 compares the Raman spectra after excitation with a laser wavelength of 514 nm, normalized to the G-band intensity, of the HOPG samples irradiated with low (LD) and high (HD) doses, for two impinging energies (400 keV (LE) and 1000 keV (HE)). D band and D′ band (Figure 1a) are depicted separately from the 2D band (Figure 1b) in order to stress out the observed changes for each mode. We should mention that each spectrum is the average of several spectra measured in the three sets of samples, in order to improve the statistics of the results.

The Raman spectrum of pristine HOPG is well known [5,8,30]. The high structural ordering of this material is reflected in two main peaks at 1580 cm^−1^ (first-order or G band) and 2690 cm^−1^ (second-order or 2D band). The G band arises from the degenerate in-plane E_2g_ mode at the center of the Brillouin zone (denoted LO), while the 2D band corresponds to the harmonic of an in-plane transverse mode, close to the K point of the zone boundary (denoted TO) [5,8,30].

Figure 1a denotes the initial disorder in the carbon matrix before the irradiation (pristine sample), detected by the presence of the D band of very low intensity, around 1367 cm^−1^. In the irradiated samples, different degrees of increasing disorder appear, depending on the combination of dose and energy used for the irradiation experiments. A new feature at 1630 cm^−1^ in the irradiated samples emerges (Figure 1a), which corresponds to the D′ band, a mode that is usually absent in perfect structures but becomes active in graphitic materials with defects because of the double-resonance Raman scattering processes that originate in electronic π–π* transitions [5,8–9,31–33]. Thus, the generation of structural defects induced by H^+^ at different doses and energies becomes evident in our experiments. It is accepted that the D band involves a double-resonance process that activates a TO phonon (“inter-valley” defect-induced electron–hole). On the other hand, the D′ band is activated by a similar double-resonance process that activates an LO phonon (“intra-valley”). Despite the fact that the D band and the D′ band have been widely reported [5,8–9,30–33] there is no agreement about the potential mechanisms that contribute to their intensity or shape modifications.

**Figure 2:**
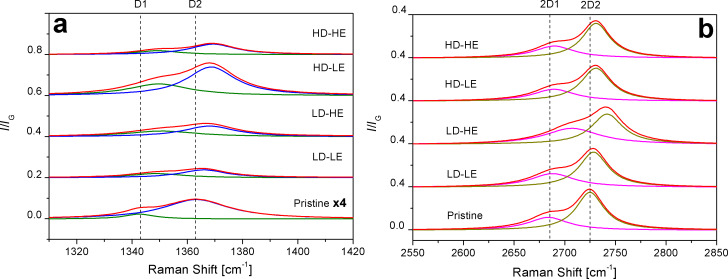
Deconvolution of a) D band and b) 2D band. The dotted vertical lines are drawn as a guide to the eye, at the peak positions in the pristine sample. Note the enlargement of the intensity scale of the pristine sample in panel a).

In order to better understand the induced modifications in the D band and the 2D band, deconvolutions into Lorentzian band shapes are depicted in Figure 2, after a careful subtraction of the baseline.

From the deconvolution of the D band (Figure 2a), we verify that it is a doublet (D_1_ and D_2_). Positions of D, D′ and 2D components, as well as the *I*_D1_/*I*_D2_ ratio, are summarized in Table 1, in order to emphasize the intensity changes in the D_1_ and D_2_ contributions.

**Table 1:** Irradiation parameters and average values of position and relative intensity for D, D′ and 2D contributions.

sample	dose [ions/cm^2^]	energy [MeV]	D_1_ position [cm^−1^]	D_2_ position [cm^−1^]	*I*_D1_/*I*_G_	*I*_D2_/*I*_G_	*I*_D1_/*I*_D2_ [%]	D′ position [cm^−1^]	2D_1_ position [cm^−1^]	2D_2_ position [cm^−1^]

pristine	—	—	1343	1363	0.006	0.024	25	—	2684	2725
LD-LE	10^14^	0.4	1349	1366	0.020	0.039	51	1623	2688	2728
LD-HE	10^14^	1.0	1350	1369	0.028	0.052	54	1628	2707	2742
HD-LE	10^16^	0.4	1351	1368	0.057	0.140	41	1626	2689	2730
HD-HE	10^16^	1.0	1349	1369	0.019	0.052	37	1627	2690	2731

The doublet of the D band is associated to the rupture of the space symmetry in each graphene layer due to the disorder originating from armchair edges, point-like defects and the formation of C–H sp^3^ bonds from C–C sp^2^ bonds [9,15,18]. It is worth noting that both D-band components are already apparent in the pristine HOPG, although with much lesser intensity. Furthermore, in this latter sample, the normalized intensity of D_2_ (*I*_D2_/*I*_G_) is 4 times larger than the normalized intensity of D_1_ (*I*_D1_/*I*_G_) before the irradiation experiments. Likewise, if we look into this behavior for the irradiated samples by inspecting the *I*_D1/_*I*_D2_ ratio, we notice that the D_2_ intensity remains larger than that of D_1_, but now its increment varies between two- and three-times *I*_D1_/*I*_G_, when varying the dose or energy. In fact, the D_2_ component almost doubles the D_1_ component (a factor of 1.95 for LD-LE and 1.86 for LD-HE), when the dose is kept at the lower value.

When keeping the dose constant at the higher value, we find that the increment of the D_2_ contribution with respect to that of D_1_ is more than 2.4-times larger (2.43 for HD-LE and 2.74 for HD-HE). This may indicate that D_2_ is mainly dominated by the irradiation dose rather than by the impinging energy. Therefore, a larger increment in the intensity of the D_2_ component is expected when the dose of H^+^ ions increases, at a fixed energy. With the same trend, while keeping the energy at a constant value (low or high), the increment of the dose produces the larger differences between the intensities of the D_1_ and D_2_ contributions (Table 1). This effect hints that shorter or stronger buckled C–C bonds or both configurations resulting from C–H bond formation [18] contribute mostly to the D_2_ component.

In Figure 2 we observe blue-shifts of the position of the D band as well as of the position of the harmonic 2D with respect to pristine HOPG. We also notice a small shift in the position of the D′ band (Table 1), taking as a reference the lower resolved peak in sample LD-LE. These shift effects are usually attributed to increments in the laser excitation energy. However, this interpretation is not appropriate for our results, since the wavelength was kept constant at 514 nm and, consequently, dispersive behavior is neglected. Thus, it can be claimed that the structural disorder is mainly due to point-like defects and armchair edges generated by the irradiation [5,9,15,34], although this latter contribution might have already be present in a small amount in the pristine HOPG, as shown in Figure 1a. Perfect zigzag edges do not contribute to the increase of the D band [5]. Regarding the identification of C–H sp^3^ bonds originating from hydrogenation of HOPG layers, a decoupling of structural disorder from hydrogenation is not possible, because the cross section of C–C sp^3^ bonds in visible Raman characterization is negligible [9,18,21]. Besides, the observed shapes of the *I*_D_/*I*_G_ ratio and the G band are consistent with those corresponding to graphite-like hydrogenated amorphous carbon with low H content (lower than 20%), according to the classification proposed by Casiraghi and co-workers [15].

In Figure 2b we also note that the 2D band appears as a doublet, and that it is less sensitive to the effect of irradiation than the D band and the D′ band. The 2D band arises from the splitting of the π and π* electronic states due to the interactions between the graphite layers. For graphene (one layer), the 2D band is a singlet, while for two layers the band appears as a quadruplet. For a material composed by more than five graphene layers, the Raman spectrum is almost the same as that of graphite, with the 2D band appearing as a doublet [30,33]. From the features of the 2D band in our Raman spectra, it is possible to conclude that (at least in the short-to-medium range) the graphene layers in the HOPG samples remain ordered along the hexagonal axis, even after irradiation.

From our results, we observe that irradiation of HOPG with H^+^ ions at 400 keV and 1000 keV, using different doses for each energy, induces a shift and broadening of the D band components with respect to pristine HOPG. This band is more sensitive to the effects of irradiation, depending on the combination of dose and energy. We do not observe a continuous evolution from low dose and low energy to high dose and high energy in our spectra, which may allow us to correlate changes in each component of the D band with a certain kind of defects or defect-like features, including changes in the hybridization state coming from the formation of C–H bonds. Therefore, we interpret our results as two different vibrational behaviors resulting from at least two different types of structural defects that involve armchair edges, point-like defects, and/or changes of C–C sp^2^ bonds due to formation of C–H bonds.

**Figure 3:**
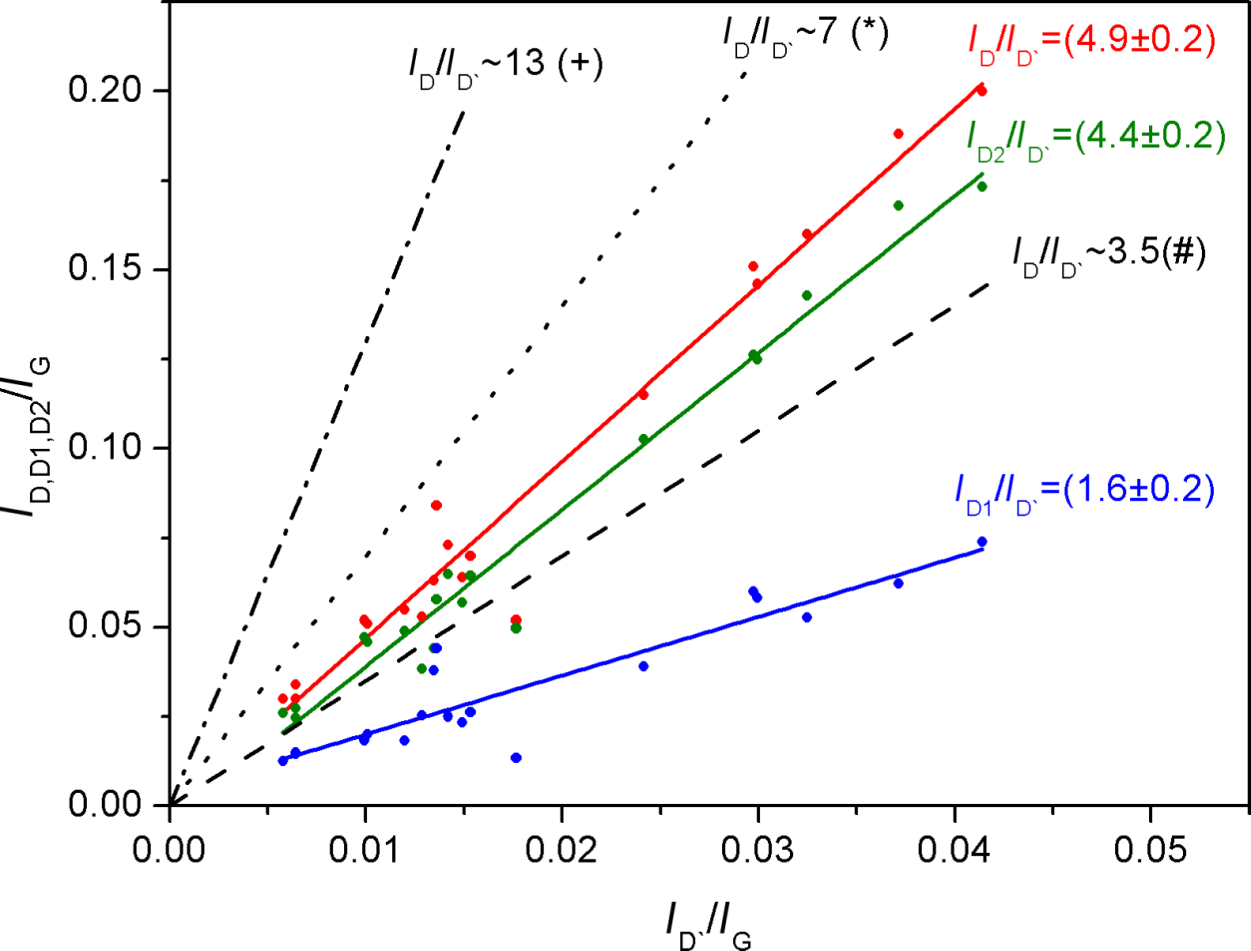
Linear dependence of the (total) *I*_D_/*I*_D′_ ratio and the (separate) contributions of *I*_D1_/*I*_D′_ and *I*_D2_/*I*_D′_ for the irradiated samples. The dots and solid lines represent the measured values in this work, while the dashed, dashed-dotted and dotted lines are taken from Eckmann and co-workers [35]. The symbols +, * and # refer to sp^3^-hybridized bonds, vacancies, and boundary defects, respectively.

Numerous studies have shown that the relative intensity of the D band to the G band, *I*_D_/*I*_G,_ increases with increasing disorder resulting from structural and topological defects as well as H implantation in hydrogenated samples. Tuinstra et al. [8] introduced a method for determining the average crystal domain size by considering the intensity ratio *I*_D_/*I*_G_. Likewise, Ferrari [9] proposed that the evolution of Raman spectra can be fitted by a phenomenological model in agreement with the amorphization trajectory for graphitic nanocrystallites. The authors pointed out that the *I*_D_/*I*_G_ intensity ratio depends on the mean nanocrystallite size and the phase of graphite [9]. According to this model, the different factors that influence the Raman spectrum are: the ratio between sp^2^ and sp^3^ bonds in the sample, sp^2^-phase clustering, rings and chains, and bonding disorder [8–9,27–28]. In addition, Casiraghi et al. [15] investigated the multi-wavelength Raman spectra of a variety of hydrogenated amorphous carbon materials, which allowed them to estimate values for their bond structure, hydrogen content and mechanical properties. A remarkable conclusion is that UV Raman spectroscopy allows for the identification of not only structural and topological disorder, but also of C–H and C–C sp^3^ bonds [15], a task which is not feasible with visible Raman spectroscopy, because the cross section of sp^3^-hybridized bonds for visible light excitation is negligible. Hydrogenated graphene Raman spectra have also been investigated by Luo and co-workers [34]. The authors found up to four double-resonant scattering processes contributing to the D band, which originated from the hydrogen atom coverage, but no correlation between different kinds of defects and the shape of the sub-bands was given. In this sense, Eckman et al. [35] identified the type of defects generated in different HOPG samples and were able to relate the corresponding *I*_D_/*I*_G_-vs-*I*_D′_/I_G_ ratios to different kinds of defects. They found that *I*_D_/*I*_D′_ ≈ 13 is related to sp^3^-hybridized sites in fluorinated graphene, *I*_D_/*I*_D′_ ≈ 7 refers to vacancies in ion-bombarded graphene, and *I*_D_/*I*_D′_ ≈ 3.5 to boundary-like defects in graphite. According to this model, we assessed the trend of *I*_D_/*I*_D′_ for our experiments and show the result in Figure 3.

We have considered the total D ratio *I*_D_/*I*_D′_ as well as both separate contributions *I*_D1_/*I*_D′_ and *I*_D2_/*I*_D′_ and find that each sub-band represents a different group of defects. Based on these results for graphene, we claim that D_1_, with the lowest slope (1.6 ± 0.2), could be related to boundary-like defects in HOPG, while D_2_, with a slope of (4.4 ± 0.2), could be associated to vacancies and armchair edges [5,12,36], and to the contribution of shorter and/or stronger buckled C–C bonds originating from the formation of C–H bonds due to the ion irradiation process. It has been reported that the D band of graphene is more sensitive to defects than that of graphite [12,18,36], possibly because of a layer effect, in which decreasing intensities of the D band are observed for samples with increasing number of graphene layers. Keeping this in mind, we can explain the lower slopes of *I*_D_/*I*_D′_ with respect to Eckman’s work [35]. Thus, applying the *I*_D_/*I*_D′_ criterium and comparing our results for HOPG with those reported by Eckman for graphene, we find an additional support to our previous assignment regarding the dependence of the D_2_ band on the dose. In fact, the contribution of C–C sp^2^ bonds coming from the formation of C–H bonds increases with a higher irradiation dose. This type of defect would contribute mainly to the D_2_ sub-band, explaining why we observe that D_2_ is mainly affected by the irradiation dose rather than by the impinging energy.

In order to correlate structural defects with ferromagnetic ordering in our samples, magnetization measurements were carried out.

### Magnetic characterization

**Figure 4:**
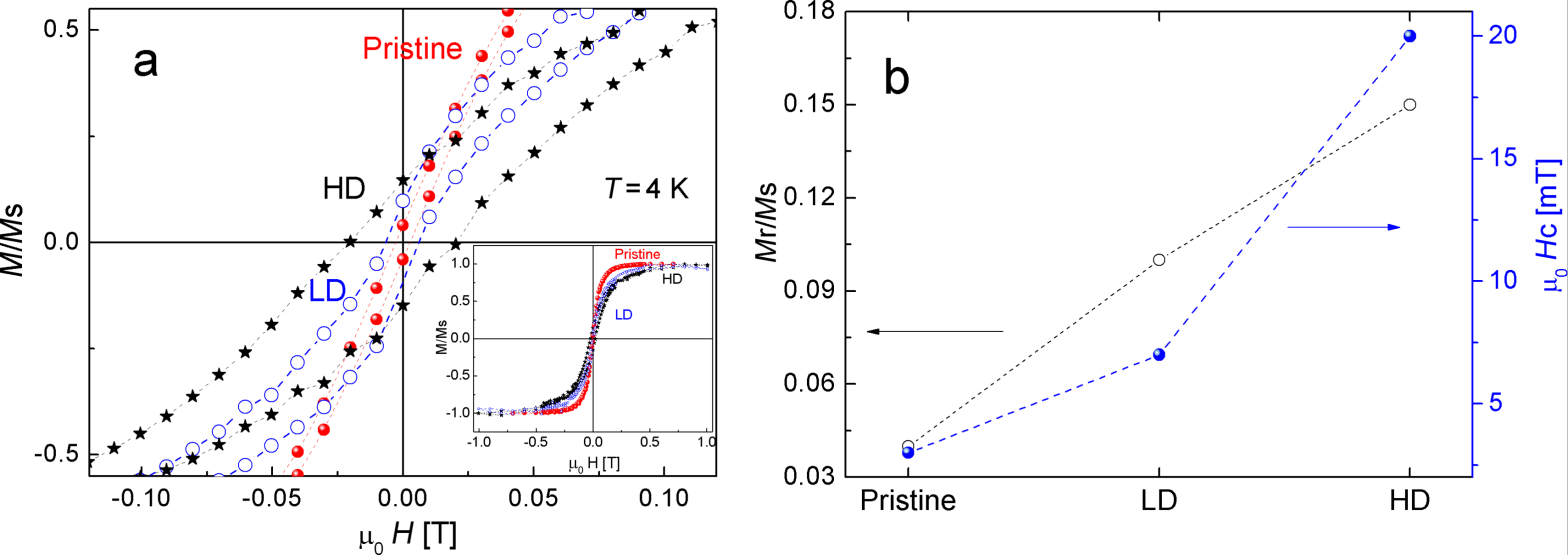
a) Hysteresis loops of pristine HOPG and irradiated samples with low (LD) and high (HD) H^+^ doses, in the zero-field region, after subtracting the diamagnetic contribution. The inset shows the complete loops, measured with a maximum applied field of 1 T, perpendicular to the hexagonal *c*-axis. b) Normalized remanence (left axis) and coercivity (right axis).

In view that the defects responsible for the increase of the D band in the Raman spectra are related to magnetic changes in HOPG samples [37], magnetization measurements were conducted in order to attempt a more complete characterization of our samples. Hysteresis loops of pristine HOPG and the irradiated samples were measured at 4 K after Raman characterization. Figure 4a shows the normalized magnetization *M*/*M*_s_ as a function of the magnetic field *H* for samples irradiated with an energy of 0.4 MeV (LE), low and high doses, together with the pristine sample, after subtracting the diamagnetic contribution inherent to graphite. Even when no magnetic impurities were determined in the pristine HOPG within the detection limit of PIXE measurements, a small ferromagnetic contribution is noticed in this sample. This is expected in ZYB-grade HOPG and has been reported in previous papers [24,38–40].

A significant enhancement in both the normalized remanence *M*_r_/*M*_s_ and coercivity µ_0_*H*_c_ is noticed after H^+^ irradiation. The increment in these quantities is rather proportional to the irradiation dose and is larger in the HD sample, consistently with a larger defect density. Figure 4b depicts this behavior, which indicates that the larger the dose, the closer the defects and, therefore, an enhanced interaction between magnetic moments. Similar results were obtained for the samples irradiated with 1 MeV (HE), indicating that the relevant parameter for controlling the magnetic response in HOPG is the dose and not the energy of the impinging ions. This result is in agreement with other works [25,39–42], in which the authors prove that it is possible to induce magnetic ordering in graphite by controlled ion irradiation.

Some authors suggest that the most likely mechanism involved in the ferromagnetic ordering induced in H^+^-irradiated graphite is related to the structural defects produced in the volume where the ions are implanted, regardless of the nature of the impinging ions [39]. On the other hand, other researchers [43] show that defect-induced magnetism (DIM) does not simply increase with dose and that there is an optimum dose above which DIM decreases again. They further suggest that DIM in HOPG is most likely not a volume effect, but a surface effect due to hydrogen present at the surface. This could explain why DIM can be triggered with electron irradiation by the formation of hydrogen-chemisorption defects at the surface [28,43].

The defect separation for a dose of 10^16^ protons/cm^2^ is in the range of 5 nm [39]. Reported experimental results [39] showed that ferromagnetic behavior in irradiated graphite is obtained when the mean distance between the produced defects is about 2 nm. According to this data, the observed enhancement in both the normalized remanence *M*_r_/*M*_s_ and in coercivity µ_0_*H*_c_ after H^+^ irradiation evidences that an interaction between magnetic moments is promoted [25,38–41].

### AFM characterization

**Figure 5:**
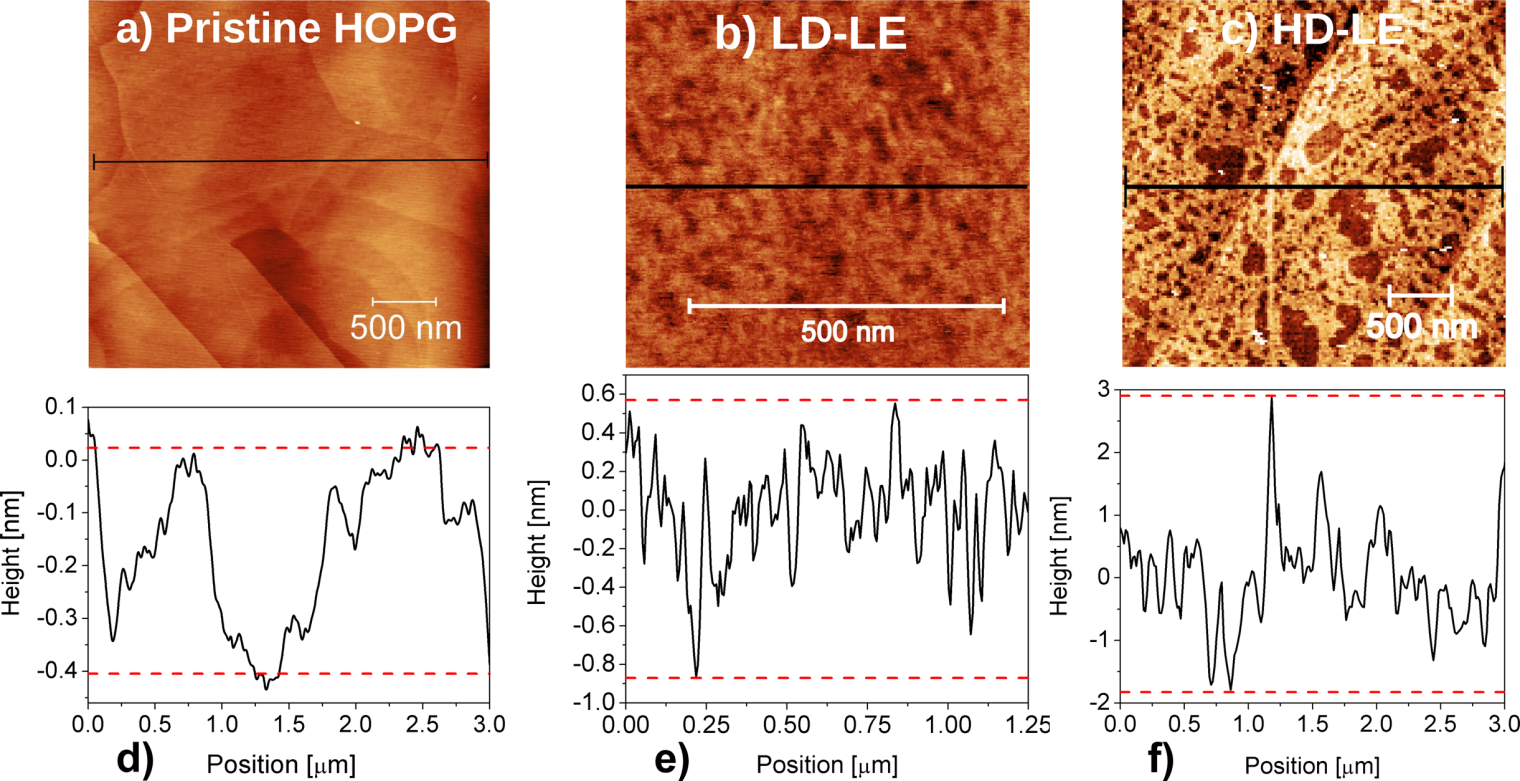
AFM images and height profiles of (a, d) pristine HOPG, (b, e) HOPG irradiated at low dose and low energy (LD-LE), and (c, f) HOPG irradiated at high dose and low energy (HD-LE).

Figure 5 shows AFM images of the HOPG surface before (Figure 5a) and after H^+^ implantation at 0.4 MeV, for low dose (Figure 5b) and high dose (Figure 5c). The corresponding height profiles are depicted in Figure 5d–f.

**Figure 6:**
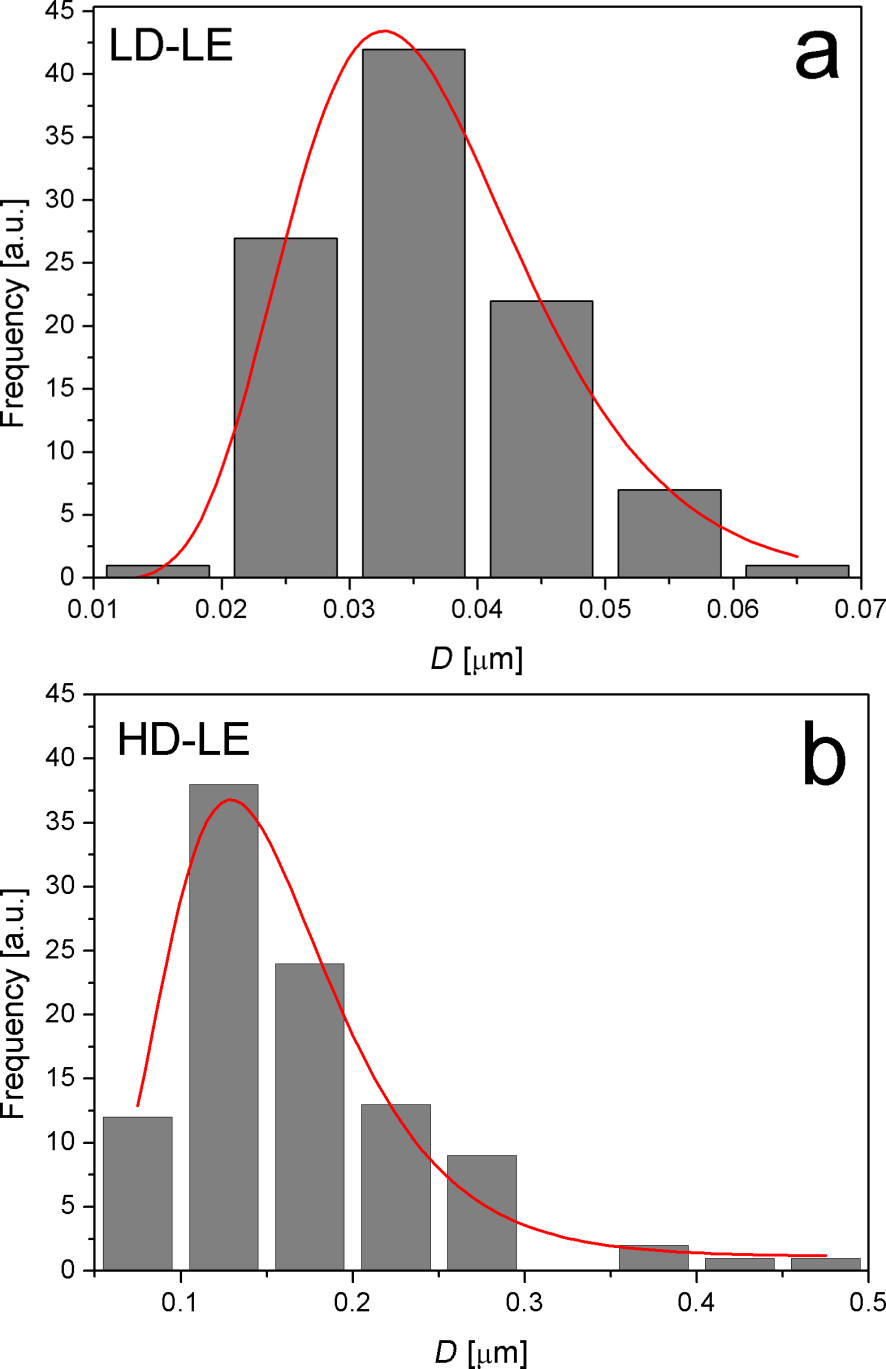
Statistical analysis of superficial defect sizes obtained from AFM images for a) low dose (LD-LE) and b) high dose (HD-LE).

A striking difference between the pristine and irradiated HOPG is the distribution of defects in both irradiated samples at a fixed energy of 400 keV. In fact, an average height of 0.4 nm was found for pristine HOPG, while average values of 2 nm and 5 nm were measured for the heights of defects produced at low dose (Figure 5e) and high dose (Figure 5f), respectively. Comparing low and high dose (LD-LE and HD-LE) in Figure 5b and Figure 5c, respectively, a strking difference in the size of the surface defects is noticed. On the other hand, the corresponding images for high-energy (1 MeV) irradiations (not shown), do not evidence surface defects that can be related to the irradiation process. This is possibly because the depth in which the defects are produced with high impinging energy is approximately four times greater than in the case of lower energy. In fact, SRIM simulations allowed us to estimate penetration depths of 3.3 μm and 12.6 μm for low and high energies, respectively. Hence, a smaller mean free path of the ions results in the case of low energy, which produces a higher defect density. The results of statistical analyses carried out to determine the average defect size in both samples are shown in Figure 6a and Figure 6b for LD-LE and HD-LE, respectively. An average defect size of 32 nm results for defects induced at the lower dose, while at the higher dose the average size is around 130 nm.

The observed defects are certainly much larger than the expected from the ion irradiation. This might be possible according to the explanation provided in [21–22], where the authors find that H^+^ irradiation leads to the accumulation of hydrogen inside the HOPG matrix, in bubbles or blisters located amongst the graphene layers. The defective topography of the irradiated samples observed in Figure 5b,c is interpreted as a consequence of the bursting of H blisters. Taking into account the work of Waqar et al. [22], we have estimated the pressure inside one of such blisters considering half ellipsoids with a mean volume given by the mean size obtained from AFM data. For sample LD-LE, we obtain *p*_LD-LE_ ≈ 2 × 10^6^ Pa, and for sample HD-LE it is *p*_HD-LE_ ≈ 4 × 10^7^ Pa. Considering that typical values of tensile and compressive strengths of graphite are of the order of 10^7^ Pa [22,44] we conclude that the estimated pressures are strong enough to be the cause of the blisters bursting on the surface of samples HD-LE and LD-LE.

## Conclusion

H^+^ ion irradiation with 0.4 MeV and 1 MeV, at two different doses, has been used to introduce disorder in HOPG. The use of Raman spectroscopy allowed us to reach a deeper insight on the expected set of defects contributing to the changes observed in each of the two components of the D band. We find that the effect in the increment of *I*_D2_/*I*_G_ with respect to *I*_D1_/*I*_G_ is larger when increasing the dose. This result indicates that the D band, and in particular the D_2_ component, is strongly dominated by the dose rather than the H^+^ penetration depth (energy). This result discloses that a larger contribution of defects, originating from a rupture of C–C sp^2^ bond symmetry through the formation of C–H sp^3^ bonds, is expected in the D_2_ component. Our results are also in good agreement with the expected magnetic response after H^+^ ion irradiation, which is effective for enhancing both the remanence and coercivity of the pristine HOPG. After Raman and SQUID characterization, AFM measurements were performed. A high density of surface defects was observed, probably due to the bursting of the stored H_2_ molecules inside the HOPG matrix. This last effect may not only hint a potential path of patterning, but also contribute to the current interest of developing carbon-based materials for hydrogen storage.
